# Risk of infection and transmission of SARS-CoV-2 among children and adolescents in households, communities and educational settings: A systematic review and meta-analysis

**DOI:** 10.7189/jogh.11.05013

**Published:** 2021-07-17

**Authors:** Omar Irfan, Jiang Li, Kun Tang, Zhicheng Wang, Zulfiqar A Bhutta

**Affiliations:** 1 Centre for Global Child Health, The Hospital for Sick Children, Toronto, Canada; 2 Vanke School of Public Health, Tsinghua University, Beijing, China; 3 Institute for Global Health & Development, the Aga Khan University, Karachi, Pakistan

## Abstract

**Background:**

There is uncertainty with respect to SARS-CoV-2 transmission in children (0-19 years) with controversy on effectiveness of school-closures in controlling the pandemic. It is of equal importance to evaluate the risk of transmission in children who are often asymptomatic or mildly symptomatic carriers that may incidentally transmit SARS-CoV-2 in different settings. We conducted this review to assess transmission and risks for SARS-CoV-2 in children (by age-groups or grades) in community and educational-settings compared to adults.

**Methods:**

Data for the review were retrieved from PubMed, EMBASE, Cochrane Library, WHO COVID-19 Database, China National Knowledge Infrastructure (CNKI) Database, WanFang Database, Latin American and Caribbean Health Sciences Literature (LILACS), Google Scholar, and preprints from medRixv and bioRixv) covering a timeline from December 1, 2019 to April 1, 2021. Population-screening, contact-tracing and cohort studies reporting prevalence and transmission of SARS-CoV-2 in children were included. Data were extracted according to PRISMA guidelines. Meta-analyses were performed using Review Manager 5.3.

**Results:**

Ninety studies were included. Compared to adults, children showed comparable national (risk ratio (RR) = 0.87, 95% confidence interval (CI) = 0.71-1.060 and subnational (RR = 0.81, 95% CI = 0.66-1.01) prevalence in population-screening studies, and lower odds of infection in community/household contact-tracing studies (odds ratio (OR) = 0.62, 95% CI = 0.46-0.84). On disaggregation, adolescents observed comparable risk (OR = 1.22, 95% CI = 0.74-2.04) with adults. In educational-settings, children attending daycare/preschools (OR = 0.53, 95% CI = 0.38-0.72) were observed to be at lower-risk when compared to adults, with odds of infection among primary (OR = 0.85, 95% CI = 0.55-1.31) and high-schoolers (OR = 1.30, 95% CI = 0.71-2.38) comparable to adults. Overall, children and adolescents had lower odds of infection in educational-settings compared to community and household clusters.

**Conclusions:**

Children (<10 years) showed lower susceptibility to COVID-19 compared to adults, whereas adolescents in communities and high-schoolers had comparable risk. Risks of infection among children in educational-settings was lower than in communities. Evidence from school-based studies demonstrate it is largely safe for children (<10 years) to be at schools, however older children (10-19 years) might facilitate transmission. Despite this evidence, studies focusing on the effectiveness of mitigation measures in educational settings are urgently needed to support both public health and educational policy-making for school reopening.

As of 5 April 2021, there have been 131.0 million confirmed COVID-19 cases and nearly 2.8 million confirmed deaths globally [1]. The response in countries worldwide has gone from an initial stage of strict lockdowns and business closures to variable periods of relaxation with social distancing, use of face masks and hand hygiene, and now vaccination roll outs for adults. During this period, daycare centers, schools and educational institutions were closed initially and then reopened at different stages. This has however, been a bone of much contention across the world with various countries adopting different measures. The global spread of variants of concern now threatens to reverse progress and disrupt the opening of the economy, commerce and education.

School closures are understandable. Children play an important role in the transmission of some respiratory infectious diseases and may suffer from more severe outcomes than adults, such as influenza [2,3], rendering school closures an effective public health policy in reducing the spread and influence of these diseases. This is especially true in novel pandemics where pharmaceutical interventions, such as vaccines, are not immediately available and delaying disease spread is a priority [3-6]. However, children and adolescents under 19 years of age comprise a small proportion of total reported COVID-19 cases (1%-10%) [7-9]. This group has been reported to present with a milder clinical course compared to adults infected with SARS-CoV-2, with more favorable outcomes in general [7,9-11].

To date, there is much controversy concerning the benefits of the ongoing and future closure of schools and other educational institutions in controlling the COVID-19 pandemic, as limited data on transmission of COVID-19 in educational settings is available [12-16]. It is of equal importance to evaluate the risk of susceptibility and transmission in children who are often asymptomatic or mildly symptomatic carriers, that may incidentally transmit SARS-CoV-2 in both educational and community settings, especially with the third wave of COVID-19 and newer variants spreading in many Countries crippling the health care system and economy.

We undertook a systematic review of the infection and transmission rates and risks of SARS-CoV-2 in children and adolescents in household, community and educational settings since the beginning of the pandemic, to help in understanding policy responses for safe school reopening for children of various ages.

## METHODS

This systematic review is reported in accordance with the Preferred Reporting Items for Systematic Reviews and Meta-Analyses (PRISMA) reporting guidelines.

In this review, we focused on the following review objectives:

1-What is the overall risk of infection in children and adolescents compared to adults (>19 years) from population screening and contact-tracing studies?

2-What are the odds of being an infected contact in children and adolescents compared to adults (>19 years) in educational settings?

3-What is the risk of infection for children and adolescents in educational settings in comparison to that in communities?

### Literature search

To investigate the risk of SARS-CoV-2 infection and transmission in children and adolescents and their potential contribution to transmission in various settings, we searched for national and subnational prevalence studies, and contact-tracing studies (CTS) from community/household clusters and educational settings.

Data for the review were retrieved from PubMed, EMBASE, Cochrane Library, WHO COVID-19 Database, China National Knowledge Infrastructure (CNKI) Database, WanFang Database, Latin American and Caribbean Health Sciences Literature (LILACS), Google Scholar, and “Living Evidence of COVID-19” (a database updated daily with published articles from PubMed and EMBASE and preprints from medRixv and bioRixv) covering a timeline from December 1, 2019 to April 1, 2021. Preprints from ChinaXiv (http://www.chinaxiv.org/home.htm) were also searched. Complementary searches were conducted by manually searching the national public health websites, and the John Hopkins Humanitarian Health Resource. The reference lists of all retrieved articles were examined as well. There was no language restriction applied for the search. The search terms applied for each research question and the specific search strategies for PubMed and other databases are provided in Table S1 in the **Online Supplementary Document****.**

The search results from various databases were uploaded into Covidence Systematic Review Software (Veritas Health Innovation 2016, Melbourne, Australia) for screening.

### Inclusion and exclusion criteria

We included population screening studies investigating the age-specific prevalence of SARS-CoV-2 infections, contact-tracing and cohort studies reporting the incidence and attack rate (number of infections secondary to a suspected close contact) of children (0-9 years) and adolescents (10-19 years old) compared to adults, case series presenting direct evidence of COVID-19 cases transmitted by SARS-CoV-2 positive children compared to adults, and data from national public health websites and official government reports, when available. We excluded review articles, opinions, viewpoints and communication letters (if not presenting data on number of infections or attack rate of SARS-CoV-2) and modeling studies were also excluded. Studies that did not report the number of infections or attack rate of SARS-CoV-2, and studies with possible duplications of cases (eg, overlapping time periods within the same institutions/cities/countries) were also excluded.

### Study screening

Two review authors independently reviewed each title and abstract from the search results. Upon obtaining the full text, two reviewers independently screened the full text and decided whether to include or exclude the study, in accordance with the criteria specified previously. Any disagreements were resolved by independent review by a third author.

### Data extraction

The following data were extracted from each study using standardized data abstraction forms: authors, country, study type, study period and its relationship with the epidemic curve in the country/area and school closure/reopen status, study setting (household, community, daycare, primary or secondary school; other mitigation measures if any), case definition (index case, primary case, secondary case), testing methods, contact-tracing methods, sampling method, number of infected children and/or adults (specified whether or not student-contacting staff) and total number of students and staff in the educational setting (or reported attack rate).

### Meta-analysis and qualitative synthesis

For each dichotomous outcome, the weighted mean prevalence and 95% confidence interval (CI) was calculated. The meta-analyses were performed using Review Manager 5.3 adopting the random-effects models. Pooled risk ratios (RR) between children and adults were presented in both national and subnational prevalence studies with disaggregation into active infection and past infection indicated by PCR testing and antibodies seroprevalence, respectively. The pooled odds ratios (OR) of children being infected in households were presented and disaggregated by children (<10 years) and adolescent (10-19 years), and school operational status (open/partially open or closed) in the region/Country. The odds of contracting infection in children compared to adults in schools and daycare centers were also analyzed. Total number of children and adolescents tested and diagnosed with COVID-19 were computed separately for communities and educational settings to calculate the odds ratio (OR) of risk of infection in educational settings compared to community settings. Statistical heterogeneity across studies was evaluated by calculating the I^2^ statistic. I^2^ values equal to or above 50% were considered as “significant” heterogeneity in this study. Additionally, the χ^2^ test for heterogeneity was performed and the forest plot was visually inspected to assess the degree of overlap between the CIs of included studies. The characteristics, biases, and results of the included studies were summarized narratively. For studies included but not eligible for meta-analysis due to a lack of sufficient data, we also qualitatively synthesized the results to present a full picture.

### Assessment of methodological quality and risk of bias

Two independent reviewers assessed each included study for methodological quality. A quality assessment tool was adopted from the National Heart, Lung, and Brain Institute (NHLBI) and Research Triangle Institute International [17] for observational studies, where are as the quality of prevalence studies was assessed using a critical appraisal checklist for prevalence studies [18]. Study quality was scored on basis of clear study objectives, case definition, consecutive inclusion of cases, sample sizes, comparability of included patients, measurement of outcomes, length of follow-up, and appropriately defined statistical methods and results. However, studies were not excluded based on study quality. Studies with score 6-8 were considered to be good quality, 4-5 considered fair quality and <4 considered poor quality.

## RESULTS

**Figure 1:**
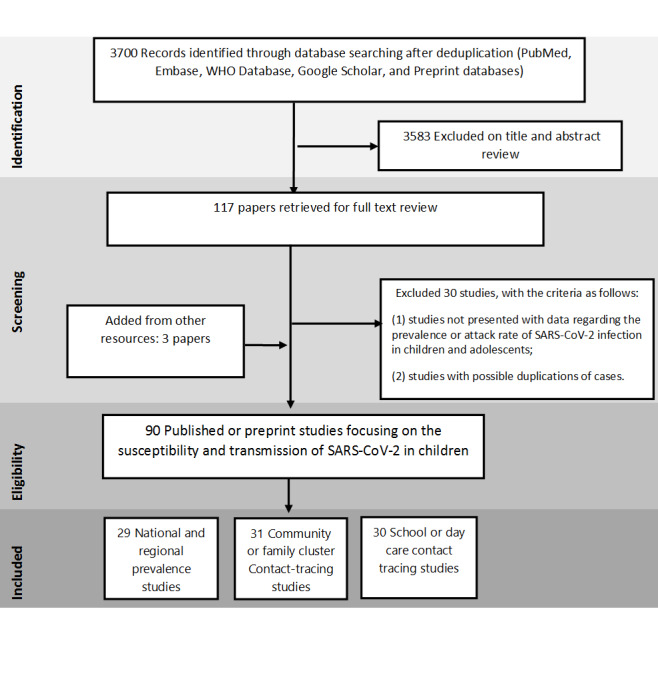
PRISMA flow diagram of study selection process.

The systemic literature search yielded 3700 results during the search dates. Of these, 117 studies were examined in full text and 90 were included in the final analysis after addition of 3 studies from other resources (**Figure 1**). The characteristics of included studies were summarized in Table S2 in the **Online Supplementary Document**. Thirty studies were excluded because they either presented overlapping data, provided little age-disaggregated data for children, or were commentaries, editorials or reviews with no empirical data.

### The overall risk of SARS-Cov-2 infection among children and adolescents in comparison to adults

To investigate the overall risk of SARS-Cov-2 infection among children and adolescents, we included 60 studies, of which 29 were population-screening studies [19-47] and 31 were CTS [48-78].

**Figure 2:**
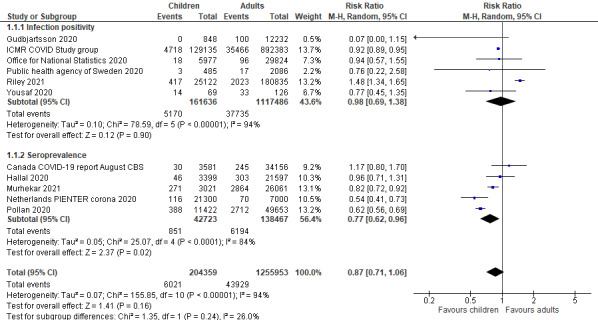
Pooled risk ratio of SARS-Cov-2 infection in children vs adults in national surveillance, disaggregated by infection positivity and seroprevalence.

**Figure 3:**
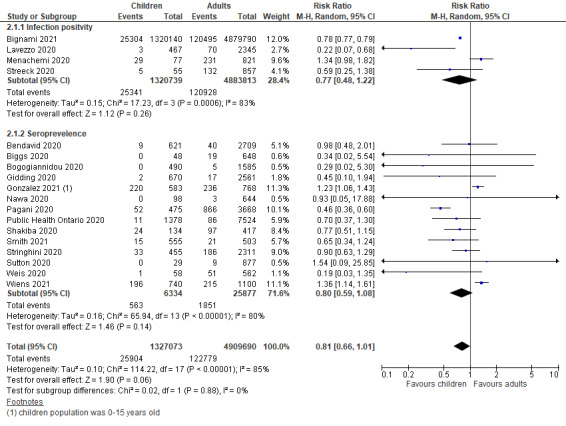
Pooled risk ratio of SARS-Cov-2 infection in children vs adults in subnational surveillance, sub-grouped into infection positivity and seroprevalence.

The prevalence of COVID-19 in children and adolescents (<20 years of age) were reported in 11 national and 18 subnational surveillance studies. Among them, six were from low- and middle-income countries (LMICs). Compared to adult populations, a comparable risk of SARS-CoV-2 infection was observed in children and adolescents in both national (RR = 0.87, 95% CI = 0.71-1.06) and subnational (RR = 0.81, 95% CI = 0.66-1.01) surveillance studies, as shown in **Figure 2** and **Figure 3**. When disaggregated by testing methods (ie, RT-PCR vs serological test), children and adolescents showed a similar lower risk of past infection from seroprevalence data in national (RR = 0.77, 95% CI = 0.62-0.96) studies but insignificant effect in subnational studies (RR = 0.80, 95% CI = 0.59-1.08). The risk of active infection was lower compared to adults but insignificant in both national studies (RR = 0.98, 95% CI = 0.69-1.38) and subnational surveillance studies at point estimate level (RR = 0.77, 95% CI = 0.48-1.22).

**Figure 4:**
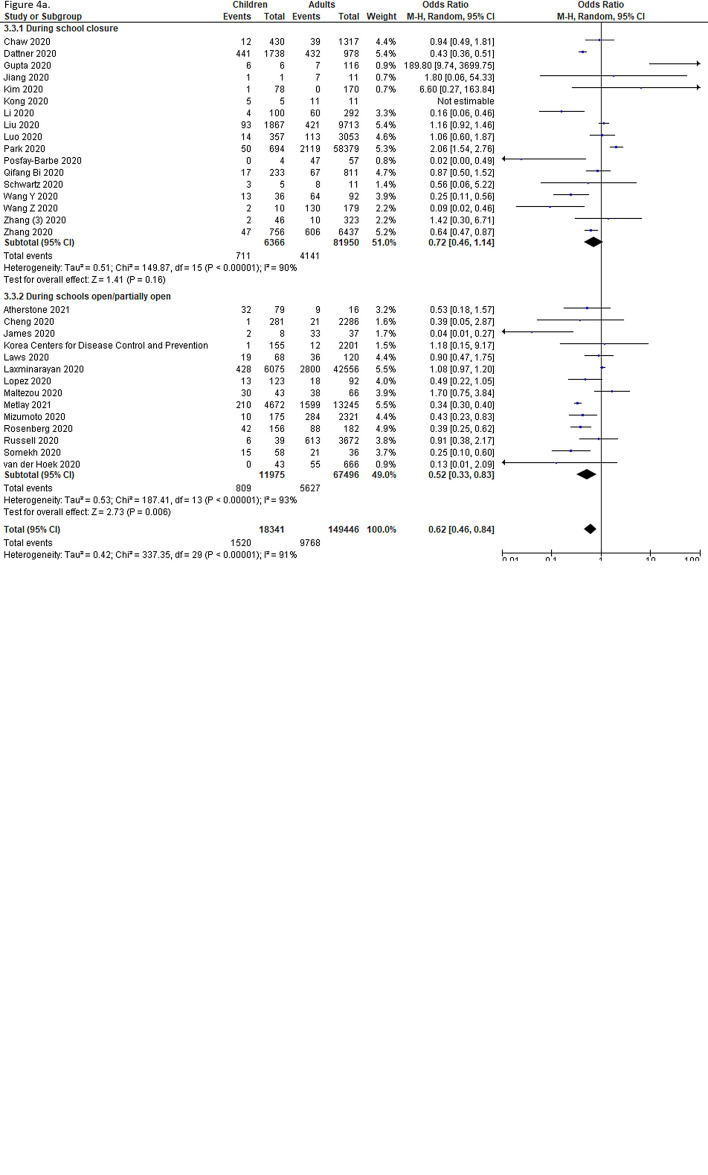
Pooled odds of children and adolescents being an infected contact in community and household family clusters **Panel A.** Odds of children and adolescents being infected vs adults, by school status. **Panel B.** Odds of children and adolescents being infected vs adults, by age group (subset of studies in Panel A).

Thirty-one studies undertook contact-tracing in community, household and family clusters, of which, 12 were from LMICs. The pooled odds of secondary attack in children and adolescents was significantly lower than that in adults (OR = 0.62, 95% CI = 0.46-0.84), with high heterogeneity (I^2^ = 0.91) (**Figure 4**, Panel A). When further disaggregated by the schools’ operational status (ie, school closure vs school fully or partially open) during the study period, both children and adolescents were found to have lower risk of infection than did adults when schools were fully or partially open (OR = 0.52, 95% CI = 0.33-0.83), but no significant effect during school closures (OR = 0.72, 95% CI = 0.46-1.14).

In a subgroup analysis of CTS (based on 18 out of the 31 studies) in which age-disaggregation was possible, we found that the pooled OR for SARS-CoV-2 infection among children compared to adults was 0.57 (95% CI = 0.37-0.87), suggesting a significantly lower risk of secondary attack in this population at the community and household level. However, a comparable risk of secondary attack was observed among adolescents (OR = 1.22, 95% CI = 0.74-2.04) (**Figure 4**, Panel B).

### Infection and transmission of COVID-19 among children and adolescents in educational settings compared to adult teachers and staff

Thirty studies conducted in educational settings were included [12-15,57,79-103], among which six studies were cross-sectional studies and the remaining 24 were contact-tracing or cohort studies. Upon checking the availability of sufficient data for comparison of children vs adults, 24 studies were included in the meta-analysis.

**Figure 5:**
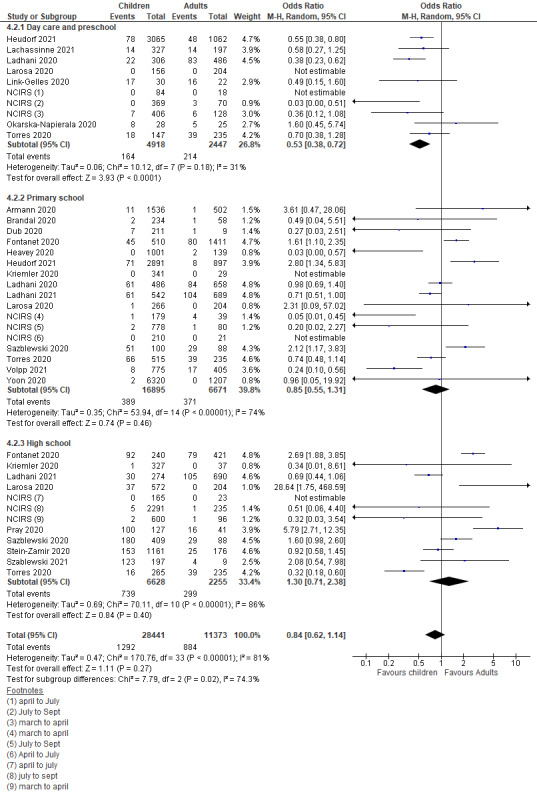
Pooled odds ratios for children and adolescent contracting infection compared to adults, by educational setting.

The pooled estimate of the included studies suggested that children and adolescents appeared to have a lower though statistically insignificant risk of secondary attack in school settings when compared to adults (OR = 0.84, 95% CI = 0.62-1.14) (**Figure 5**). Subgroup analysis also suggested significant lower odds of infection among children attending daycare centers/preschools (OR = 0.53, 95% CI = 0.38-0.72), but insignificant effect in primary schools (OR = 0.85, 95% CI = 0.55-1.31) compared to the adult staff. However, high-school students had comparable risk of infection to adults (OR = 1.30, 95% CI = 0.71-2.38).

### Risk of contracting SARS-CoV-2 infection among children and adolescents in schools compared to community settings

Using the existing evidence from both community-based studies and studies conducted in educational settings, we further calculated the pooled odds ratios for contracting infection among children and adolescent in educational settings vs communities and household-clusters. When total number of children and adolescents tested and diagnosed with COVID-19 in the two settings were compared, children observed lower odds of infection (OR = 0.53, 95% CI = 0.38-0.75) in schools compared to community and households, which was consistently observed on disaggregation by age; children (<10 years) (OR = 0.45, 95% = 0.39-0.51); adolescents and high-schoolers (OR = 0.63, 95% CI = 0.56-0.72) (Figure S1 in the **Online Supplementary Document**).

### Study quality assessment

The majority of included studies were considered of good or fair quality based on the scores generated by using quality assessment tools (Table S3 in the **Online Supplementary Document**). Out of the 29 population prevalence studies, 28 were of good quality while one was of fair quality. Twenty-five out of 31 contact-tracing studies were of good quality while six were of fair quality. For studies conducted in educational settings, eight were of fair quality and the remaining 22 were of good quality.

Studies were primarily downgraded for inadequate sample size and unclear description of study setting. However, potential biases were noted for some of the included studies, which could negatively affect their quality (eg, low response rate from the study population in contact-tracing studies [15,81,83,85], only symptomatic cases receiving tests [12,14,79,86,104]).

## DISCUSSION

This systematic review provides a comprehensive assessment of COVID-19 risk of infection and transmission in children and adolescents compared to adults in household, community and educational settings and in the relationship of age and school contexts with risks of transmission. Consistent with previous reviews [105,106], we found an overall lower risk of infection among children and adolescents (0-19 years) in households and communities compared to adults. In educational settings, children attending daycare, preschool and primary school presented a lower risk of infection than that of adults.

Our review has several important strengths. Compared to existing reviews [105,106], most of which were conducted at an earlier stage of the pandemic, the present review provided the most up-to-date evidence of this research question. We have undertaken several pre-specified sub-group analyses as per data availability. The subgroup comparisons included assessment of active infection (PCR test), past infection (blood serology), school operational status and differential effects by age groups. It was also important to assess if the risk of community transmission was affected by school closure. Using a broad search strategy implemented in English, Chinese and Spanish databases, we summarize evidence from 90 studies from 31 different countries. We also attempted to reduce possible overlap in cases to prevent duplication. Compared to a previous systematic review by Viner et al [105], we report almost thrice the number of studies with disaggregation of analyses by age and settings. This review is primarily limited by the large heterogeneity across studies and the lack of uniform age- and test-specific evidence for transmission in different study settings. Lastly, the evidence of COVID-19 infection in children is rapidly evolving; therefore, evidence from this review should be cautiously interpreted and regularly updated. This review does not include modelling studies, which can forecast future transmission scenarios but under various assumptions about disease transmission and immunity [107].

Currently available epidemiological data have revealed two unique features of pediatric COVID-19 cases: a relatively low prevalence in this population and milder clinical features compared to adult patients [9,108]. Several studies and reviews have studied children and adolescents’ susceptibility to SARS-CoV-2 infection and their role in transmission in different settings. Viner et al. examined studies on the prevalence of SARS-CoV-2 infection in children and young people (<20 years), and found that the pooled odds ratio of being infected among children vs adults was 0.56 (95% CI: 0.37-0.85) with substantial heterogeneity (I^2^ = 95%) [105]. Goldstein et al [109] reviewed data on detection of SARS-CoV-2 infection in different settings and suggested a significantly lower susceptibility of infection for children (<10 years of age) compared to adults. There was some evidence of robust spread of SARS-CoV-2 in secondary and high-schools (eg, high seroprevalence of anti-SARS-CoV-2 antibodies among high-school students in northern France [83], and an outbreak in an Israel high-school [13]), while the spread seemed to be more limited in primary schools [12,14,15,82,88-91]. Xu et al. conducted a living systematic review and reported that the SARS-CoV-2 infection attack rates were 0.15% (95% CI = 0%-0.93%) among students and 0.70% (95% CI = 0%-3.56%) among school staff, respectively [110]. These findings are largely consistent with the primary finding of the present study that children are not as susceptible to SARS-CoV-2 infection as adults, and while children are known to be “super spreaders” for influenza [111] and measles viruses [112], they play only a limited role in SARS-CoV-2 transmission in various settings.

Symptomatic patients have a lower SARS-CoV-2 cycle threshold (Ct) values, which corresponds to higher viral RNA levels. SARS-CoV-2 Ct values have been found to be almost linearly inversely correlated with its transmission [113]. Furthermore, a meta-analysis reported risk of asymptomatic transmission is significantly lower than that of symptomatic transmission (relative risk = 0.58; 95% CI = 0.34-0.99) [114]. To contextualize, these findings might suggest that children may be less likely to transmit SARS-CoV-2 due to their lower prevalence of symptomatic and severe presentation during the infection [115].

School closures are an effective public health mitigation measure in reducing the community transmission of many respiratory infectious diseases, such as influenza [2,3], however, current evidence on the effectiveness of school closures in curbing the COVID-19 pandemic is inconsistent. Large experiences from Australia, USA and England demonstrated low transmission rates in schools and early childhood education services when these facilities were still open [90,91,116]. However, Auger et al. conducted a US population-based observational study between March 9 and May 7, 2020 and found that school closure was associated with a significant decline in the incidence of COVID-19 [117]. Majority of the school linked index cases report none or only a small number of secondary cases [91,93]. Reports investigating outbreaks have demonstrated a higher transmission by school-age children to other students or teachers, particularly when the mitigation measures were inadequately implemented in schools [104]. Reports from Sweden [118] and US [119] suggest a comparable increased risk of transmission from teacher to students and other staff members. These highlight the significance of focusing COVID-19 prevention protocols and vaccination strategies for the teachers, which may indirectly protect students who might not be immediately prioritized in the vaccine rollout. In the present study, we find overall that opening educational establishments may not predispose children and adolescents to a higher risk of SARS-CoV-2 infection compared to adults. On the contrary, children and adolescents were found to have more than 2-fold greater risk of infection in household and community settings than in schools. The school attendance may serve as a protective factor, which reduces children’s chances of community contacts in a relatively isolated environment during school hours. It may also be attributable to the effective infections control measures applied in schools introduced by global and national guidelines.

Importantly, prolonged school closures have also been found to have negative impacts on the educational and social development of children including increasing mental disorders, worsening nutrition, lack of physical activities, substance abuse, child violence and abuse [106,120-124]. Lessons from the 2013-2016 Ebola pandemic suggested that youth, and young girls in particular, of poor households saw the largest increase in permanent school dropouts post-Ebola [125,126]. The disruption of education is particularly harmful to young children who are in the most sensitive window of learning, as the early education loss could permanently affect the development of one’s foundational skills [127]. Alternative educational opportunities such as online distance learning may not be available to poorer or marginalized populations and are non-existent in many LMICs [128].

The decision to reopen schools is understandably a delicate balance between various factors, including the incidence of COVID-19 cases in the community, the concerns and choices of the parents and the public, the school-based mitigation strategies in place including vaccinations for teachers and the availability of resources. It is recommended that schools should only be reopened when the prevalence of COVID-19 at the community level is under a relatively safe threshold [129].

Safe reopening of schools is not possible without proper mitigation plans and strategies in place. Some of the measures, which are suggested by the present guidelines, include repeat testing, avoiding crowded/close contact environments, social distancing, wearing facial coverings, maintaining hand hygiene, and some protective measure of classrooms and environment, including limiting classroom size and ensuring adequate ventilation including open air classes where feasible [130]. Despite these recommended actions, there are major challenges in evaluating the effectiveness of such guidelines. It is even more challenging to ensure the most effective interventions to be properly implemented in schools. Mitigation strategies at schools may incur a considerable financial cost. For instance, it was estimated that an additional 20 billion USD would be needed for the nationwide implementation of recommended school-based mitigation strategies in the US [131].There is currently limited data on and much need for collating evidence from safe school reopening strategies and experience across the world.

Given the highly contagious nature of SARS-CoV-2 and the new variants, a expanding vaccine eligibility for children and adolescents and addressing it’s hesitancy is the most effective strategy for returning children to schools [116]. While some countries have prioritized vaccination of school teachers and staff to reduce occupational transmission, the evidence of effectiveness of vaccination strategies in adolescents is just emerging [132] and trials are being ramped up in younger children [133]. Given the potential serious complications of COVID-19 infection in subsets of children [134], vaccination research and implementation in children must be prioritized across the world.

## Additional material

Online Supplementary Document
