# Supplementary material for: Risk of infection and transmission of SARS-CoV-2 among children and adolescents in households, communities and educational settings: A systematic review and meta-analysis
Source: J Glob Health. 2021 Jul 17;11:05013. doi: 10.7189/jogh.11.05013 (PMC8285769; doi:10.7189/jogh.11.05013)

Appendix Table S1: PubMed Search strategy

Search terms used for SARS-CoV-2 infection

(“COVID-19”[tw] OR “2019-nCoV”[tw] OR “severe acute respiratory syndrome coronavirus 2” [tw] OR 2019-nCoV [tw] OR “Wuhan coronavirus” [tw] OR “SARS-CoV-2” [tw] OR “2019 novel coronavirus” [tw] OR “COVID-19 virus” [tw])

Search terms used for prevalence and community contact tracing studies

(“transmission”[tw] OR “transmission” [mh] OR “Susceptibility”[tw] OR “epidemiology”[tw] OR “contact tracing”[tw]))

Search terms used for infection transmission in educational settings

(“child day care centers” [mh] OR “child day care centers” [tw] OR “daycare centers for children” [tw] OR "nurseries, infant" [mh] OR “nurser* [tw] OR Schools [mh] OR school*[tw] OR “primary school” [tw] OR “primary schools”[tw] OR “secondary school”[tw] OR “secondary schools”[tw])

Appendix Table S2. Characteristics of Studies included in the meta-analysis (n=77)

Study	Study design	component of review	Country	Setting	Timeline	Methods
**National and regional prevalence studies of community and household transmission**						
Canadian Blood Services, et al.	national surveillance database	prevalence	Canada	Nationwide seroprevalence survey	May 9 to Jun 18, 2020	Canadian Blood Services and Canada's COVID-19 Immunity Task Force collected blood samples for antibodies against SARS-CoV-2 of Canadian residents.
DF. Gudbjartsson, et al.	national surveillance database	prevalence	Iceland	Targeted testing and population screening	Jan 31 to Apr 4, 2020	Testing people living in Iceland who were at high risk for infection, and population screening using two strategies: issuing an open invitation to 10,797 persons and by sending random invitations to 2283 persons
Murhekar, et al.	national surveillance database	prevalence	India	Household serosurvey among individuals aged 10 years or older in the same 700 villages or	Between Aug 18 and Sept 20, 2020	Participant serum samples were tested for the presence of SARS-CoV-2 specific IgG antibodies on the Abbott Architect i2000SR automated analyser using the Abbott SARS-CoV-2 IgG assay

				wards within 70 districts in India		
Hallal, et al.	national surveillance database	prevalence	Brazil	Nationwide seroprevalence survey in 133 sentinel cities in 26 Brazilian states.	May 14-21, 2020	Randomly selected households visited. Finger-prick rapid serology test was employed.
Yousef, et al.	national surveillance database	prevalence	Saudi Arabia	All patients presenting to health care facilities who were positive for COVID-19 across all regions	Mar 1 to Mar 31, 2020	Data were extracted from the Health Electronic Surveillance Network database
ICMR COVID study group, et al.	national surveillance database	prevalence	India	The system covered the entire population of India throughout 426 testing centers nationwide	Jan 22 to Apr 30, 2020	The reported testing results are based on qRT-PCR
Netherlands PIENTER corona, et al.	nationwide population study	prevalence	Netherlands	Nationwide seroprevalence survey	Mar 31 to Apr 13, 2020	Samples were collected from a randomly chosen subset of municipalities across the Netherlands.
Office for National Statistics	nationwide population study	prevalence	England	Representative sample of 35,801 individuals in England.	Apr 26 to Jun 27, 2020	Cases were identified by home self-sampling using nasopharyngeal swabs. Repeated surveys carried out each week.
Pollan et al.	nationwide population study	prevalence	Spain	50 Spanish provinces and the two autonomous cities with municipalities.	Apr 27 to May 11, 2020	Households were selected from municipal rolls using two-stage random sampling stratified by province and municipality size, with all residents

						invited to participate, aiming at investigating the seropositivity for SARS-CoV-2 in the non-institutionalized Spanish population.
Public health agency of Sweden	nationwide population study	prevalence	Sweden	Two nationally-representative surveys	Apr 21-24 and May 25-28, 2020	Participants invited by email and participants performed home self-sampling using nasopharyngeal swabs.
Riley, et al.	nationwide population study	prevalence	England	Nationally-representative survey	6th January 2021 to 15th January 2021	swab-positivity prevalence from REACT-1 with mobility data based on the GPS locations of individuals using the Facebook mobile phone app
Bendavid, et al.	seroprevalence survey of a county	prevalence	US	Santa Clara County	April 3-4, 2020	Investigators tested county residents for antibodies to SARS-CoV-2 using a lateral flow immunoassay. Participants were recruited using Facebook advertisements targeting a sample of individuals living within the county.
Bignami, et al.	Population based surveillance data	prevalence	Canada	Provincial data for Montreal, Toronto and Calgary	August 18,2020 to January 21, 2021	Official counts of COVID-19 cases (defined as positive real-time reverse transcription– polymerase chain) by age were obtained from provincial

						reporting jurisdictions. For Montréal, they were extracted from aggregate counts of COVID-19 cases released in weekly reports by the Direction régionale de santé publique4. For Toronto and Calgary, individual-level case report data were available from, respectively, Ontario Health5 and Alberta Health6
Biggs, et al.	population-based serologic survey	prevalence	US	DeKalb and Fulton counties in metropolitan Atlanta	Apr 28 to May 3, 2020	A two-stage cluster sampling design was used to randomly select 30 census blocks in each county
Bogogiannidou, et al.	cross sectional survey (repeated at monthly intervals)	prevalence	Greece	Nationwide laboratory network	Mar to Apr, 2020	Blood samples were collected by using the leftover sampling methodology from patients visiting laboratories for check-up. They applied a geographically stratified sampling plan based on regional units (NUTS level 3).
Lavezzo, et al.	citywide surveillance	prevalence	Italy	City of Vo in Italy	Feb 20, 2020	There were two surveys conducted less than 2 weeks apart, to investigate population exposure to SARS-CoV-2

						before and after the lockdown.
Menachemi, et al.	statewide population prevalence study	prevalence	US	State of Indiana	May 2–3, 2020	The study population was randomly selected from a list of Indiana residents derived from tax returns. State databases were cross-checked for recent contact information, and institutionalized and deceased persons were removed. Stratified random sampling was conducted among all persons aged ≥12 years using Indiana's 10 public health preparedness districts as sampling strata. After the study was announced, 15,495 participants were contacted by the state health department.
Nawa, et al.	population-based seroprevalence survey	prevalence	Japan	1000 households in Utsunomiya City and Greater Tokyo	Jun 14 to Jul 5, 2020	A random sample of 1000 households were approached.
Pagani, et al.	population-based seroprevalence survey	prevalence	Italy	The town of Castiglione d'Adda, Lombardy	Jun, 2020	The entire population (all ages) invited to participate
Public Health Ontario	statewide seroprevalence	prevalence	Canada	Province-wide serosurveillance of Ontario	Mar 27 to Jun 30, 2020	The Ontario COVID-19 Serosurveillance System used residual specimens (blood, serum or plasma

						left<br>over after diagnostic testing) to test for antibodies against SARS-CoV-2 infection
Smith, et al.	subnational seroprevalence	prevalence	USA	St. Louis, Missouri, USA	Between 14 April 2020 and 12 May 2020	Five hundred three adult and 555 pediatric serum/plasma samples were collected from patients presenting to Barnes-Jewish Hospital or St. Louis Children's Hospital
González, et al	subnational seroprevalence	prevalence	Nicaragua	León, Nicaragua	Between September and October 2020	We contacted the household members of cohort children (both adults and children) and offered participation. We collected baseline demographic and health history data and collected blood from all participants for baseline SARS-CoV-2 serology.
Wiens, et al.	subnational seroprevalence	prevalence	Sudan	Juba, South Sudan	Between August 10 to September 11, 2020	Household-based cross-sectional serosurvey in Juba, South Sudan.<br>We quantified IgG antibody responses to SARS-CoV-2 spike protein receptor-binding domain and estimated seroprevalence using a

						Bayesian regression model accounting for test performance.
Shakiba, et al.	Subnational seroprevalence	prevalence	Iran	5 counties in Guilan province, northern Iran	Apr, 2020	A multistage cluster random sampling approach and telephone recruitment of head of household was adopted for this population-based seroprevalence study
Streeck, et al.	Subnational seroprevalence	prevalence	Germany	Carnival held on 15 February in Gangelt.	Mar 30 to Apr 7, 2020	A random sample of 600 households was invited to participate. 1007 individuals from 405 households participated
Stringhini, et al.	Subnational seroprevalence	prevalence	Switzerland	Population-representative survey of Geneva city	Apr 6 to May 9, 2020	SEROCoV-POP study is a population-based study of former participants of the Bus Santé study and their household members. A series of 12 consecutive weekly serosurveys among randomly selected participants from a previous population-representative survey, and their household members aged 5 years and older was conducted.
Sutton, et al.	subnationalseroprevalence	prevalence	US	Eighty-six facilities participating in CDC's Influenza-like Illness Surveillance	May 11 to Jun 15, 2020	The seroprevalence of infection with SARS-CoV-2 in Oregon was investigated by a cross-

				Network and Oregon's Electronic Surveillance System		sectional, population-based convenience sample for SARS-CoV-2 immunoglobulin G (IgG) antibody testing.
Weis, et al.	subnationalseroprevalence	prevalence	Germany	Neustadt-am-Rennsteig community in Thuringia	May 12-16, 2020	All community households were invited. 71% (626/883) of the households in the community were enrolled.
Gidding, et al.	Subnational seroprevalence	prevalence	Australia	Sydney, Australia	April to June 2020	Cross-sectional, involving de-identified residual blood specimens from public and private laboratories and Australian Red Cross Lifeblood collected April to June 2020, sampled by geographic location across 10-year age groups.
**Contact tracing studies of community and household transmission**						
Metlay, et al.	contact tracing study	transmission	USA	conducted within the Mass General Brigham system, a large integrated hospital and ambulatory care network based in Boston, Massachusetts.	between March 4 and May 17, 2020.	retrospective cohort study of COVID-19 risk among exposed children and adults in households where an index case of COVID-19 was diagnosed between March 4 and May 17, 2020.
Maltezou, et al.	contact tracing study	transmission	Greece	We studied family clusters diagnosed in three reference	February 26 (first COVID-	Family clusters were identified through the

				laboratories for SARS-CoV-2 (two in Athens and one in Thessaloniki) where most cases were diagnosed.	19 case diagnosed in Greece) through May 3, 2020	national registry of SARS-CoV-2 infections. Families with at least one child were included in the study.
Atherstone, et al.	contact tracing study	transmission	USA	High school wrestling tournament in Florida, USA	Between December 2020–January 2021	Contact tracing of tournament attendees. The tournaments included 10 participating high schools from three counties.
Chaw, et al.	contact tracing study	transmission	Brunei	All 71 initial cases in Brunei, which arose following a religious event, with cases detected after Mar 9 2020.	March, 2020	Detailed contact tracing by Ministry of Health, with RT-PCR testing of all reported contacts. All contacts were quarantined for 14 days and retested if symptomatic.
Cheng, et al.	contact tracing study	transmission	Taiwan	Data for the first 100 cases in Taiwan from the Taiwan CDC	Jan 15 to Mar 18, 2020	100 Index cases and 2761 close contacts were investigated.
Dattner, et al.	contact tracing study	transmission	Israel	The city of Bnei Brak	Jan to May, 2020	The households in the city of Bnei Brak, Israel were identified, where all household members had been tested by PCR and 1 or more members had positive findings. These households were identified through the Israeli COVID-19 database.

Gupta, et al.	contact tracing study	transmission	India	All the pediatric index patients and their primary contacts (immediate family members) in the month of May with a history of inter-state travel were included in the study	May 2020	The immediate familial members (parents/siblings) of asymptomatic travelers from states with a high incidence of COVID-19 were identified as primary contacts and were tested as per feasibility
James, et al.	contact tracing study	transmission	US	Investigation after a 3-day church event in Arkansas on March 8.	March 6–8, 2020	Contact tracing of church attendees were performed.
Jiang, et al.	contact tracing study	transmission	China	A family cluster was reported to hospital in Henan Province	Jan to Feb, 2020	12 patients involved in a family transmission of SARS-CoV-2 were investigated.
Kim, et al.	contact tracing study	transmission	South Korea	Reviewed the data on household members who were under surveillance after contacting a pediatric COVID-19 index case who reported to the National Notifiable Disease Surveillance System.	January to 6 April 2020	Contact tracing of household members was conducted. Secondary cases were defined as household contact with a pediatric COVID-19 index case, occurring at least 1 day after but within 14 days from the last point of exposure
Kong, et al.	contact tracing study	transmission	China	five family clusters with COVID-19 from Jinan city, China	January 2020 to the end of March 2020	Contact tracing of family clusters of patients presenting to Jinan

						Infectious Diseases Hospital
Korea Centers for Disease Control and Prevention	contact tracing study	transmission	South Korea	Contact tracing of the first 30 cases in Korea	Jan 24 to Mar 10, 2020	Demographic, epidemiological, and early clinical information were retrieved from COVID-19 reporting and surveillance data from Korea CDC.
Laxminarayan, et al.	contact tracing study	transmission	India	Data were collected from 2 provinces in India (Tamil Nadu and Andhra Pradesh)	Mar to Jun, 2020	The index cases identified from state registries and their contacts traced by public health agencies in each state were investigated.
Laws, et al.	contact tracing study	transmission	US	the US Centers for Disease Control and Prevention (CDC) conducted a household transmission investigation in Milwaukee, Wisconsin, and Salt Lake City, Utah, metropolitan areas	March to May 2020	Index case patients and household contacts were interviewed by using standard questionnaires that captured demographics, medical history, previous SARS-CoV-2 testing, and symptoms since the index case patient's illness onset. At study enrollment (day 0), the CDC team visited the household to collect blood and respiratory specimens.
Li, et al.	contact tracing study	transmission	China	Patients and their household contacts in 2 cities in Hubei province.	Jan to Feb, 2020	105 index patients and 392 household contacts were enrolled. Index cases identified from 2 hospitals.

Liu, et al.	contact tracing study	transmission	China	COVID-19 cases from Guangdong Province	Jan to Mar, 2020	11,580 contacts of confirmed COVID-19 cases in Guangdong Province were investigated. All contacts were tested by RT-PCR of throat swabs taken every a few days during their 14-day quarantine period.
Lopez, et al.	contact tracing study	transmission	US	17 child care facilities (day care facilities and day camps for school-aged children) in Salt Lake County	Apr 1 to Jul 10, 2020	Contact tracing data collected from three COVID-19 outbreaks in child care facilities through Utah's National Electronic Disease Surveillance System.
Luo, et al.	contact tracing study	transmission	China	Close contacts of persons infected with SARS-CoV-2 in Guangzhou, China	between 13 January and 6 March 2020	A cell phone database based on the movements of the users for close contact tracing on public transportation was used. By measuring and recording proximity events between individuals, it can immediately trace close contacts of diagnosed cases upon case confirmation.
Mizumoto, et al.	contact tracing study	transmission	Japan	All confirmed cases through contact tracing from Japan.	Jan to Mar, 2020	A total of 313 domestically acquired cases have been confirmed by performing RT-PCR to 2496 close contacts of suspected cases.

Park, et al.	contact tracing study	transmission	South Korea	Both household and non-household contacts of index COVID-19 cases were identified and monitored	Jan 20 to Mar 27, 2020	59,073 contacts of 5,706 COVID-19 index patients were monitored for an average of 9.9 (range 8.2–12.5) days.
Posfay-Barbe, et al.	contact tracing study	transmission	Switzerlan d	A university hospital in Geneva	Mar 10 to Apr 10, 2020	all patients <16 years old with SARS-CoV-2 infection were identified by means of the Geneva University Hospital's surveillance network. Then their clinical data were retrieved and contacts were followed.
Rosenberg, et al.	contact tracing study	transmission	US	New York State excluding New York City	Mar 20, 2020	The contacts of 229 initially confirmed cases were followed.
Russell, et al.	contact tracing study	transmission	Japan	Passengers of the Diamond Princess cruise ship	February 20, 2020	Data from passengers of the Diamond Princess cruise ship were analyzed.
Schwartz, et al.	contact tracing study	transmission	US	A 3-week family gathering of five households	July to Aug, 2020	Four state health departments and CDC investigated a COVID-19 outbreak that occurred during a 3-week family gathering of five households.
Somekh, et al.	contact tracing study	transmission	Israel	Mayenei Hayeshuah Medical center in the city of Bnei Brak	June 21–27, 2020	Thirteen family clusters were investigated.
van der Hoek, et al.	contact tracing study	transmission	Netherland s	National surveillance data	Jan to April 2, 2020	Contact-tracing was undertaken for all cases

				from two Dutch systems		registered in HPZone. Contact infection status identified through linkage to the main national surveillance database, suggesting that only symptomatic secondary cases were included.
Wang, et al.	contact tracing study	transmission	China	Union hospital of Wuhan, Hubei Province	Feb 20, 2020	85 households with SARS-CoV-2 infected members in Wuhan were investigated to determine the transmission rate.
Wang, et al.	contact tracing study	transmission	China	Contacts of infected patient presented at Third People's Hospital of Shenzhen	Jan 11 to Feb 29, 2020	After the onset of illness in an infected family member, all the other family members were investigated.
Zhang, et al.	contact tracing study	transmission	China	Contact-tracing surveillance system	Jan 28 to Mar 15, 2020	Asymptomatic COVID-19 cases were found mainly through close contact screening, clustered epidemic investigations, follow-up investigation of infection sources, and active surveillance of key populations with travel or residence history in areas with continuous transmission of COVID-19 in China and abroad.
Zhang, et al.	contact tracing study	transmission	China	2 cities in China	Jan 16 to Mar 1, 2020	1245 contacts reported by 636 study participants in Wuhan and 1296 contacts

						reported by 557 participants in Shanghai. Close contacts were identified through contact tracing of confirmed cases and placed under medical observation for 14 days.
Wang, et al.	contact tracing study	transmission	China	335 people in 124 families from Beijing	Feb 28 to Mar 27, 2020	335 people in 124 families in which there was at least one laboratory confirmed COVID-19 case were investigated.
**Transmission in educational settings**						
Ladhani, et al	cohort study	transmission	England	131 schools in England	June–December, 2020	SARS-CoV-2-positive staff and students were invited to enrol in a household transmission study, in which all household members were swabbed by the sKIDs investigation team and had blood samples taken for antibody testing 4–6 weeks later.
Szablewski, et al.	cohort study	transmission	USA	High school sleep away camp in Georgia	June 10, 2020, to July 1, 2020	Case patients were defined as attendees who had a state- or self-reported positive viral test result or met the CSTE clinical criteria without test information.

Lachassinne, et al.	cohort study	transmission	France	Children and staff who attended one of 22 daycare centres during a nationwide lockdown in France were included in this	Between March 15 and May 9, 2020	Cross-sectional, multicentre, seroprevalence study. Hospital staff not occupationally exposed to patients with COVID-19, or to children, were enrolled in a comparator group. The primary outcome was SARS-CoV-2 seroprevalence in children, daycare centre staff, and the comparator group.
Heudorf U, et al.	cohort study	transmission	Germany	From the 35th to the 52nd calendar week, 274 index cases from 143 daycare centers and 75 schools were reported.	August to December 2020	The index cases from daycare centers and schools were isolated, and the CPs were offered PCR testing for SARS-CoV-2 on a voluntary basis - regardless of whether symptoms suggestive of SARS-CoV-2 occurred or not.
Volpp K, et al.	cohort study	transmission	USA	A New Jersey grade 9–12 boarding school with 520 full-time resident students, 255 commuter students, and 405 faculty and staff members.	August 20–November 27, 2020	During August 20–November 27, RT-PCR tests were performed on 8,955 saliva specimens from 405 faculty and staff members and 12,494 nasal swab specimens from 775 students.

Stefanie Desmet, et al.	cohort study	transmission	Belgium	84 children attending 8 different daycare centers in Belgium	Mar 2020	The current study was embedded in the nasopharyngeal (NP) carriage study that started since 2016 to monitor changes in pneumococcal serotypes in children between 6 and 30 months of age, attending daycare centers.<br>Sample collected during the period of March 2-12 were analyzed.
Chen Stein-Zamir, et al.	contact tracing after an outbreak	transmission	Israel	High school	May 26, 2020	After an outbreak in a high school after school reopening, the complete school community was tested for secondary SARS-CoV-2 infection via RT-PCR.
Juan Pablo Torres, et al.	cross-sectional study after an outbreak	seroprevalenc e	Chile	School community	May 4-19, 2020	After a SARS-CoV-2 outbreak in a large school community, the entire community was placed under quarantine. A home-delivery, self-administered, IgM/IgG antibody test and survey were conducted in a classroom stratified sample of students and all staff.
Timothee Dub et al.	retrospective cohort study, contact tracing	transmission	Finland	2 primary schools	Mar 20, 2020	Close school contacts and families of school cases

						were tested for SARS-CoV-2 infection.
Arnaud Fontanet et al.	retrospective cohort studies, contact tracing	transmission	France	High schools	Mar 30 to Apr 4, 2020	Students and staff of high schools exposed to SARS-CoV-2 in Feb and Mar in a city north of Paris were enrolled. Participants completed a questionnaire covering history of fever and/or respiratory symptoms since Jan 13, 2020 and had blood tested for the presence of anti-SARS-CoV-2 antibodies.
Arnaud Fontanet et al.	retrospective cohort studies, contact tracing	transmission	France	Primary schools and training team	Apr 28-30, 2020	Pupils, their parents and relatives, and staff of primary schools exposed to SARS-CoV-2 in Feb and Mar in a city north of Paris were enrolled. Participants completed a questionnaire covering history of fever and/or respiratory symptoms and had blood tested for the presence of anti-SARS-CoV-2 antibodies.
Jakob P. Armann et al.	cohort study	seroprevalenc e	Germany	13 secondary schools in eastern Saxony	May 25 to Jun 30, 2020	1538 Students grade 8-11 and 507 teachers participated in the study, in which their blood samples were collected for testing anti-SARS-CoV-2 IgG.

Laura Heavey et al.	contact-tracing study	transmission	Ireland	Primary and secondary schools	Mar 20, 2020	All SARS-CoV-2 notifications to Public Health Department were screened to identify children and adults who had attended the school setting. Close and casual contacts of confirmed cases were followed up and referred for RT-PCR testing if they developed any symptoms consistent with COVID-19.
Chee Fu Yung, et al.	contact tracing study	transmission	Singapore	Preschools and secondary school	Feb to Mar, 2020	In Feb and Mar 2020, comprehensive nationwide surveillance and contact tracing identified 3 potential SARS-CoV-2 seeding incidents in 3 separate educational settings. All close contacts were placed under quarantine and received tests for SARS-CoV-2 infection either based on symptoms or based on the contact history.
Nicole E. Brown, et al.	contact tracing study	transmission	US	Schools	Mar 10-13, 2020	A teacher was diagnosed with COVID-19 after returning from Europe. Students exposed to the teacher in the classroom were invited to participate

						in the study. 21 (18%) students volunteered and received RT-PCR tests for SARS-CoV-2.
Christine M. Szablewski et al.	contact tracing study	transmission	US	An overnight camp in Georgia	Jun 20, 2020	58% attendees received SARS-CoV-2 testing after an index case was identified and secondary attack rate was calculated based on the available results.
Laura L. Blaisdell, et al.	Contact tracing study	transmission	US	Four overnight camps in Maine	Jun to Aug 2020	Attendees of these camps were screened for COVID-19 before and after their arrival. 3 asymptomatic cases were identified. The remaining attendees were followed up and tested for any secondary transmission.
I W. Pray et al.	Contact tracing	transmission	US	An overnight summer school retreat	Jul 2 to Aug 11, 2020	After two students at the retreat received positive SARS-CoV-2 test results, PCR testing and serologic tests were offered to attendees.
Ruth Link-Gelles, et al.	Contact tracing study	transmission	US	Child care program in Rhode Island	Jun 1 to Jul 31, 2020	Possible child care-associated COVID-19 cases were reported to the Department of Health and secondary transmission were investigated.

National Centre for Immunization Research and Surveillance (NCIRS)	prospective cohort study	transmission	Australia	Early child education centers (ECEC) and schools	Jan 25 to Apr 9, 2020	NCIRS has been conducting surveillance of SARS-CoV-2 transmission in educational settings throughout the 2020 school year in New South Wales. Close contacts of index cases were identified and tested for secondary cases.
NCIRS	prospective cohort study	transmission	Australia	ECEC and schools	Apr 10 to Jul 3, 2020	NCIRS has been conducting surveillance of SARS-CoV-2 transmission in educational settings throughout the 2020 school year in New South Wales. Close contacts of index cases were identified and tested for secondary cases.
NCIRS	prospective cohort study	transmission	Australia	ECEC and schools	Jul 4 to Sep 25, 2020	NCIRS has been conducting surveillance of SARS-CoV-2 transmission in educational settings throughout the 2020 school year in New South Wales. Close contacts of index cases were identified and tested for secondary cases.
Sharif A. Ismail, et al.	Cohort study	Rate of infection	England	Early year settings, primary and secondary schools	Jun 20, 2020	Public Health England initiated enhanced national surveillance following the reopening of

						educational settings. COVID-19 related situations in educational settings across England were reviewed daily and followed-up. SARS-CoV-2 infection and outbreak rates were then calculated for students and staff attending these educational settings.
Brandal LT, et al.	Contact tracing study	Transmission	Norway	Primary school	Aug 28 to Nov 11, 2020	Contacts of 13 index cases aged 5-13 years in Oslo or Viken county who had attended school within 48 hours before symptom onset or date of sampling were invited to participate in this study. Two saliva samples were self-collected on the same day, following standard protocols. Each saliva sample was analyzed for SARS-CoV-2 using PCR.
Larosa E, et al.	Contact tracing study	Transmission	Italy	Schools including infant-toddler center/preschool, elementary, middle and high schools	Sep 1 to Oct 15, 2020	All consecutive COVID-19 cases in Reggio Emilia province among children and adolescents (0-19years) who had possible exposure or contacts in school during the study period were identified. All classmates and staff who

						had contact with the index case were immediately tested and retested 14 days after the last contact with the index case if the first test was performed more than 10 days after the contact; usually only one test was performed.
Yoon Y, et al.	Contact tracing study	Transmission	Korea	Kindergartens, elementary, middle and high schools	May 20 to Jul 31, 2020	After children attending kindergartens and schools and diagnosed with COVID-19 after off-line classes started were identified, students and staff of the relevant schools and kindergartens were tested for SARS-CoV-2 infection.
Okarska-Napierala M, et al.	Contact tracing study	Transmission	Poland	Nursery	May 31 to Jun 13, 2020	After an index case of SARS-CoV-2 infection was identified, subsequent PCR testing of nursery staff, children attending the facility and family members were performed.
Kriemler S, et al.	Cohort study	Point prevalence	Switzerlan d	Primary and secondary schools	Dec 1-11, 2020	A buccal swab for PCR and a rapid diagnostic test to detect SARS-CoV-2 were taken twice 1 week apart in a cohort of children from randomly selected

						primary and secondary schools and classes.
Uylte A, et al.	Cohort study	Serological prevalence	Switzerland	Primary and secondary schools	Jun to Nov, 2020	Children from randomly selected schools and classes, stratified by district, were invited to participate in serological testing of SARS-CoV-2.
Ladhani S, et al.	Cohort study	Rate of infection	England	Preschools and primary schools	Jun 1 to mid-Jul, 2020	The COVID-19 Surveillance in School KIDs (sKIDs) study involved two arms. In the swabs arm, schools across England with at least 30 students attending for at least 4 weeks during the summer half-term were approached to take part in the study. For the serology arm, schools that were not participating in weekly swabbing were approached in five regions and serological samples were taken.

Appendix Table S3. Quality assessment of Studies included in the review (n=77)

Author name	Clear objectives	Were the participants identified suitable for the objectives of the study?	Was the sample size adequate?	Setting clearly described	Description of participants clearly defined	Were valid methods used for the identification of the condition?	Were the statistical methods well-described?
**Community contact tracing studies**							
Atherstone C 2021	1	1	0	1	1	1	1
Chaw L 2020	1	1	0	1	1	1	1
Cheng HY 2020	1	1	0	0	1	0	1
Dattner I 2020	1	1	1	1	0	1	1
Gupta N 2020	1	1	1	1	1	1	1
James A 2020	1	1	0	1	1	1	1
Jiang Y 2020	1	1	0	0	1	1	1
Kong X 2020	1	1	1	1	1	1	1
Kim J 2020	1	1	1	1	1	1	1
Korea Centers for Disease Control and Prevention 2020	1	1	0	0	1	1	1
Laxminarayan R 2020	1	1	0	0	1	1	1
Laws 2020							
Li W 2020	1	1	0	0	1	U	1
Liu T 2020	1	1	1	1	0	1	1
Lopez AS 2020	1	1	0	1	1	1	1
Luo 2020	1	1	1	1	1	1	1
Maltezou 2020	1	1	1	1	1	1	1
Metlay 2021	1	1	1	1	1	1	1
Mizumoto K 2020	1	U	1	1	0	U	1

Park YJ 2020	1	1	1	1	0	1	1
Posfay-Barbe C 2020	1	1	0	0	1	1	1
Qifang B 2020	0	1	0	0	1	1	1
Rosenberg ES 2020	1	1	1	1	1	1	1
Russell TW 2020	0	1	0	1	1	1	1
Schwartz NG 2020	1	1	0	1	1	1	1
Somekh E 2020	1	1	0	0	1	1	1
van der Hoek 2020	1	1	1	0	0	1	1
Wang Z 2020	1	1	0	0	1	0	1
Wang Y 2020	1	1	1	1	1	U	1
Yousaf AR 2020	1	1	1	1	0	1	1
Zhang J 2020	1	1	1	0	1	1	1
Zhang W 2020	1	1	0	0	0	1	1
**National and regional prevalence studies**							
Murhekar M 2021	1	1	1	1	1	1	1
González F 2021	1	1	1	1	1	1	1
Wiens K 2021	1	1	1	1	1	1	1
Gidding H 2020	1	1	1	1	1	1	1
Riley S 2020	1	1	1	1	1	1	1
Bignami S 2021	1	1	1	1	1	1	1
National Institute for Public Health	1	1	1	U	U	1	1

and the Environment 2020							
Pollan M 2020	1	1	1	1	1	1	1
Hallal P 2020	1	1	1	1	0	1	1
Biggs H 2020	1	1	0	1	1	1	1
Shakiba M 2020	1	1	1	1	1	1	1
Stringhini S 2020	1	1	1	1	1	1	1
Nawa N 2020	1	1	0	1	1	1	1
Weis S 2020	1	1	1	1	1	1	1
Pagani G 2020	1	1	1	0	0	1	1
Streeck M 2020	1	1	1	1	1	1	1
Gudbjartsson D 2020	1	1	1	1	1	1	1
Lavezzo E 2020	1	1	1	1	1	1	1
Public Health agency of Sweden 2020	1	1	1	1	1	1	1
Office for National Statistics 2020	1	1	1	1	1	1	1
Canada CBS 2020	1	1	1	1	1	1	1
Smith B 2021	1	1	1	1	1	1	1
ICMR Covid19 Study group 2020	1	1	1	1	1	1	1
Ontario study 2020	1	1	1	1	1	1	1

Bendavid E 2020	1	1	1	1	1	1	1
Bogogiannidou Z 2020	1	1	1	1	1	1	1
Menachemi N 2020	1	1	1	1	1	1	1
Sutton M 2020	1	1	0	1	1	1	1
**Studies from Educational settings**							
Desmet S 2020	1	1	0	1	1	U	0
Stein-Zamir C 2020	1	1	1	1	1	1	1
Heudorf U 2021	1	1	1	1	1	1	1
Lachassinne E 2021	1	1	1	1	1	1	1
Szablewski C 2021	1	1	0	1	1	1	1
Volpp K, 2021	1	1	1	1	1	1	1
Torres JP 2020	1	U	0	1	1	U	1
Dub T 2020	1	1	0	1	1	1	1
Fontanet A 2020	1	U	1	1	1	1	1
Fontanet A 2020	1	U	1	1	1	1	1
Armann JP 2020	1	U	U	1	1	1	1
Heavey L 2020	1	0	0	1	1	1	0
Yung CF 2020	1	0	0	1	1	1	0
Brown NE 2020	1	0	0	1	1	1	0
Szablewski CM 2020	1	0	0	1	1	1	1
Blaisdell LL 2020	1	0	0	1	1	1	1

Pray IW 2020	1	0	0	1	1	1	0
NCIRS 2020	1	1	1	1	1	1	1
NCIRS 2020	1	1	1	1	1	1	1
NCIRS 2020	1	1	1	1	1	1	1
Ismail SA 2020	1	1	1	1	1	1	1
Ladhani S 2020	1	1	1	1	1	1	1
Brandal LT 2020	1	1	1	1	1	U	1
Larosa E 2020	1	1	1	1	1	1	1
Yoon Y 2020	1	1	1	1	1	1	1
Okarska-Naplerala E 2020	1	1	U	1	1	1	1
Kriemler S 2020	1	1	1	1	1	U	1
Ulyte A 2020	1	1	1	1	1	1	1

1= Yes, 0= No, U=uncertain

Appendix Figure S1: Pooled odds ratios for children and adolescent to contract infection in educational settings compared to in communities

	Schools		Communities			Odds Ratio	Odds Ratio
**Study or Subgroup**	**Events**	**Total**	**Events**	**Total**	**Weight**	**M-H, Random, 95% CI**	**M-H, Random, 95% CI**
**5.1.1 Younger children**							
Transmission	604	16513	374	4789	49.8%	0.45 [0.39, 0.51]	
**Subtotal (95% CI)**		**16513**		**4789**	**49.8%**	**0.45 [0.39, 0.51]**	
Total events	604		374				
Heterogeneity: Not applicable							
Test for overall effect: Z = 11.81 (P < 0.00001)							
**5.1.2 Adolescents**							
Transmission	739	6628	440	2661	50.2%	0.63 [0.56, 0.72]	
**Subtotal (95% CI)**		**6628**		**2661**	**50.2%**	**0.63 [0.56, 0.72]**	
Total events	739		440				
Heterogeneity: Not applicable							
Test for overall effect: Z = 7.01 (P < 0.00001)							
**Total (95% CI)**		**23141**		**7450**	**100.0%**	**0.53 [0.38, 0.75]**	
Total events	1343		814				
Heterogeneity: $Tau^2 = 0.06$; $Chi^2 = 13.53$, df = 1 (P = 0.0002); $I^2 = 93\%$							
Test for overall effect: Z = 3.63 (P = 0.0003)							
Test for subgroup differences: $Chi^2 = 13.50$, df = 1 (P = 0.0002), $I^2 = 92.6\%$							

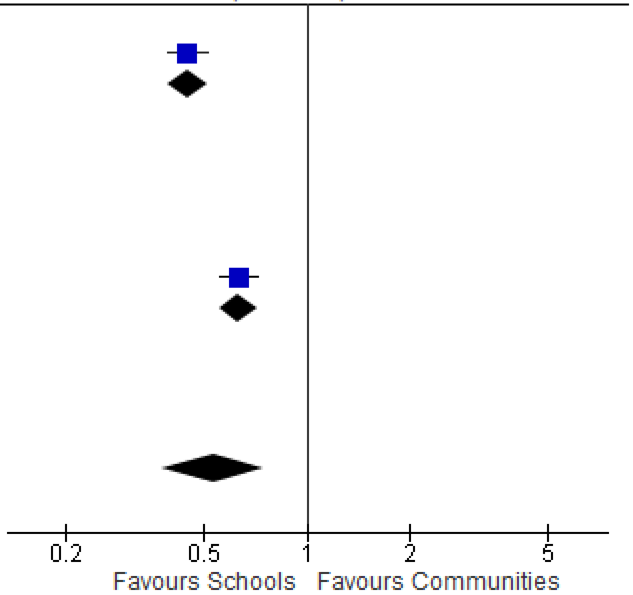

Supplement: Online Supplementary Document [file jogh-11-05013-s001.pdf]
